# Salt eustress modulates physiological responses and enhances yield, nutritional quality, and antioxidant capacity in sunflower microgreens

**DOI:** 10.1186/s12870-026-09786-y

**Published:** 2026-08-27

**Authors:** Bardees Mickky, Reham Shams Eldeen, Mustafa Elnajar

**Affiliations:** 1 Botany Department, Faculty of Science, Mansoura University, Mansoura, 35516 Egypt; 2 Botany and Microbiology Department, Faculty of Science, Arish University, Arish, 45511 Egypt

**Keywords:** *Helianthus annuus*, NaCl, Elicitation, Hormesis, Osmotic regulation, Proximate composition, Minerals, Fatty acids

## Abstract

**Background:**

Microgreens have gained attention as nutritious superfoods and as model systems for studying plant stress responses. Salt stress can elicit hormetic effects by enhancing plant growth and quality at low concentrations (eustress) while inhibiting performance at higher levels (distress).

**Methods:**

Sunflower (*Helianthus annuus* L.) microgreens were hydroponically cultivated in a completely randomized design with three vessel replicates per treatment. Seedlings were grown for ten days using root-dipping technique in five NaCl concentrations (0–100 mM) under controlled natural environmental conditions. Growth parameters, physiological status, stress biomarkers, proximate composition, mineral content, fatty acid profile, and antioxidant capacity were assessed.

**Results:**

Low stress induced by 25 mM NaCl increased shoot fresh yield (58.7%), shoot dry yield (55.4%), shoot length (48.3%), root length (25.7%), leaf area (81.4%), and specific leaf area (19.7%). Mild stress by 50 mM NaCl also improved growth, but its stimulatory effects were less pronounced. Conversely, moderate and severe stress levels (75 and 100 mM) significantly reduced these growth parameters. These responses were associated with modified physiological status indicated by photosynthesis and transpiration rates, chlorophyll content, relative water content, and water use efficiency. At 25 mM NaCl, proline content increased (5.7%), while catalase activity, lipid peroxidation, and H_2_O_2_ content did not change significantly. Higher salt levels induced marked oxidative stress through elevated lipid peroxidation and H_2_O_2_ accumulation. Nutritionally, 25 mM NaCl increased total carbohydrates (27.2%), total proteins (27.6%), Ca (11.9%), Mg (13.3%), Mn (14.3%), Zn (12.9%), and linoleic acid (5.0%). However, this low stress decreased ash content (–27.3%), total fats (–69.8%), K (–12.0%), Fe (–15.2%), palmitic acid (–9.7%), stearic acid (–12.2%), and oleic acid (–4.8%). Antioxidant profiling showed that 25 mM NaCl maximized total antioxidant activity (38.1%), DPPH-scavenging activity (6.0%), total phenols (8.8%), ascorbic acid (37.5%), and carotenoids (11.5%), while moderate and severe stress levels had opposite effects.

**Conclusion:**

Low-dose salt eustress (25 mM NaCl) can be applied as a simple and cost-effective elicitor to improve growth, nutritional value, and antioxidant capacity of sunflower microgreens.

**Supplementary Information:**

The online version contains supplementary material available at https://doi.org/10.1186/s12870-026-09786-y.

## Introduction

Microgreens are young edible seedlings with characteristic flavor, color, texture, and nutritional value. These immature seedlings can be developed from a wide range of vegetables, grains, and herbs. Microgreens are typically harvested 7 to 21 days after germination at the cotyledonary leaf stage [1]. Unlike sprouts which are germinated seeds grown entirely in dark over 3 to 5 days and consumed as a whole (seed, plumule, and radicle), microgreens require light exposure and are consumed de-rooted [2]. Because they are harvested at a highly active physiological state, microgreens possess a rich phytochemical profile of vitamins, minerals, antioxidants, as well as vital secondary metabolites such as phenolics and carotenoids [3]. In this regard, it is estimated that microgreens can have phytochemicals 4 to 40 times higher than those found in their mature counterparts [1]. According to recent estimates, the global microgreen market projected from 2.5 billion USD in 2024 to 2.7 billion USD in 2025 and is expected to reach up to 6.3 billion USD by 2033 [4].

Microgreens are often grown via smart agricultural systems using vertical or horizontal farming setups which are standards in controlled environment agriculture [5]. Due to their brief life cycle, microgreens are uniquely suited for hydroponic cultures where minimal nutrient inputs are required. Only water can be used to sustain their short growth span, as the germinating seed naturally contains all macro- and micro-nutrients needed to support development until the cotyledonary leaf stage [6]. This water-only cultivation not only produces a clean and low-cost crop, but it also establishes a highly predictable physiological model. Consequently, this baseline can enable the introduction of targeted chemical stressors to trigger adaptive metabolic pathways without the interfering variables commonly found in soil cultures. In this context, elicitation emerges as a potent biotechnological tool to induce controlled stress by adding certain elicitors to the growth medium to enhance the final harvest quality [7]. Among various abiotic elicitors, sodium chloride (NaCl) is widely recognized for its ability to cause osmotic and ionic stress, making it ideal for boosting the nutritional and antioxidant values of hydroponic crops [8].

The utilization of NaCl in soilless cultivation as a chemical elicitor follows the principles of hormesis; a biphasic biological response where low doses of an environmental stressor induce beneficial effects, while high doses result in severe physiological damage. According to this concept, abiotic stress can be categorized into eustress (positive stress) and distress (negative stress) [9]. When plants are subjected to high NaCl doses (distress), excess ions disrupt osmotic equilibrium and induce oxidative stress via the overproduction of reactive oxygen species (ROS). Furthermore, elevated ions induce deficiencies in essential nutrients and impair enzymatic reactions and hormonal signaling [10]. Conversely, when plants are subjected to low NaCl doses (eustress), they can be stimulated to activate their defense systems and upgrade their primary and secondary metabolic pathways [11]. Recent studies on different plants have documented that salt eustress could enrich their phytochemical profile with ultimate enhancement of the plant resilience as well as its nutritional and functional quality. Nevertheless, the response to salt eustress is cultivar-dependent; therefore, the selection of the appropriate cultivar is crucial to ensure successful elicitation. Also, it is vital to apply the optimal dose of eustress that causes the accumulation of protective metabolites, but not at the expense of plant growth and biomass yield [12].

Among the diverse species utilized in microgreen production, sunflower (*Helianthus annuus* L.) has gained significant attention due to its robust growth biometrics, high seed availability, and exceptional sensory properties including nutty and sweet flavor, as well as crunchy and juicy texture. Biochemically, sunflower microgreens are rich in essential fatty acids, vitamins, minerals, and phenolic compounds, making them an excellent candidate for functional food optimization [13]. Furthermore, sunflower microgreens serve as a low-calorie and high-protein food source highly compatible with vegetarian lifestyles. Also, the antioxidant potency of sunflower microgreens largely contributes to their health-promoting properties [14]. From a physiological point of view, sunflower has unique metabolic behavior because it is an oilseed plant rather than a conventional leafy green. This makes it react differently to environmental stresses [15]. Compared with other crops, sunflower is moderately tolerant to salt stress, with wide variations from one cultivar to another [16].

While the impact of salt elicitation has been intensively studied in Brassicaceae microgreens such as cabbage [17], mustard [18], and broccoli [11], limited research addresses it in Asteraceae microgreens (especially sunflower). Understanding how this specific plant balances its defensive metabolism against salinity remains a critical gap in optimizing its production. Therefore, the primary objective of the current study is to utilize the principles of hormesis to enhance the functional value of sunflower microgreens through optimized salinity elicitation. To the best of our knowledge, this study may provide the first comprehensive evaluation of salt eustress applications in hydroponically grown sunflower microgreens to optimize their biomass yield, fatty acid profile, and bioactive phytochemicals. We hypothesized that a specific level of salt eustress can induce the accumulation of protective metabolites and antioxidants without causing biomass loss. To test this hypothesis, various concentrations of NaCl were applied to determine the precise threshold where salinity can act as eustress rather than distress.

## Materials and methods

### Plant material and experimental setup

Pure strain of sunflower (*Helianthus annuus* L.), salt tolerant cultivar Giza 120, was obtained from Oil Crops Research Department, Field Crops Research Institute, Agricultural Research Center, Giza, Egypt. The cultivar was selected for its robust growth attributes and marked water use efficiency. A lot of even-sized seeds was washed thoroughly under running water and subsequently surface-sterilized for 10 min using 3% H_2_O_2_. The disinfected seeds were rinsed several times with sterile water to remove any adherent disinfectant. As a pre-conditioning step, seeds were hydroprimed for 4 h with periodic stirring to improve the rate, uniformity, and vigor of germination. During the hydropriming period, the priming water was refreshed every hour to maintain a consistent water potential and prevent the accumulation of germination-inhibiting metabolic byproducts. Following water imbibition, the seeds exhibited an approximate 75% increase in weight, accompanied by softened seed coats and a darker turgid appearance, indicating successful hydration and the onset of early metabolic activity.

Germination and early seedling experiment was conducted in a passive (non-circulating) liquid hydroponic system. Root-dipping technique was adopted as it operates without electricity and enables both controlled and efficient microgreen production. Primed sunflower seeds were spread uniformly in sprouting vessels lined with fabric mesh at a seeding rate of 45 g seeds per vessel. Each vessel (35 cm long × 25 cm wide × 10 cm deep) consisted of a top perforated tray settling into a base tray. In the root-dipping setup, the liquid in the base tray was maintained at a level that submerged the roots extending through the mesh without flooding the seeds. Seeds were covered with wet sterilized fabric and left to germinate in the dark at 28 ± 2℃ for 48 h. The vessels were vertically stacked in a completely randomized design with a 1.5 kg weight pressing on the uppermost vessel. This pressure stimulated the seedlings to push against the weight, resulting in more efficient seed coat rupture and robust hypocotyl development. The consistent pressure from the weight also helped ensure that all seeds in the vessel germinate at a similar rate, leading to a more even and uniform crop of microgreens.

On the second day, the fabric coverage was carefully removed, washed, and rewetted. The sprouts were gently washed via a top mist shower, and any contaminated seeds were discarded. The sprouts were then re-covered with the wet fabric and left in the dark with the trays vertically stacked (the stacking order was rotated daily) and compressed under the weight. On the third day, the same procedure was followed, accompanied by bottom watering with tap water to prevent radicle necrosis. On the fourth day, upon development of the cotyledonary leaves, the fabric was permanently removed, followed by top showering and refreshing the bottom irrigation. The vessels were then re-arranged horizontally in the dark for 24 h to induce etiolation, thereby promoting elongation of the hypocotyls. On the fifth day, the vessels were transferred outdoors to induce de-etiolation, with top showering and bottom irrigation. Once subjected to daylight, the sprouts turned green within 3 to 4 h.

For the subsequent five days, salt treatments were applied via bottom irrigation using 300 mL NaCl solution (0, 25, 50, 75, and 100 mM). The choice of these concentrations was based on preliminary experiments where high NaCl concentrations (up to 500 mM) were studied. Accordingly, a customized salt stress scale was established to define these treatments as low (25 mM), mild (50 mM), moderate (75 mM), and severe (100 mM) thresholds. The experiment was arranged in a completely randomized design, with three independent vessel replicates per treatment (each vessel served as a true statistical and experimental unit). To eliminate spatial effects (such as light or airflow variations), the arrangement of the vessels was randomized daily. Growth conditions were maintained with day/night temperature of 28/18 ± 2℃, relative humidity of 65 ± 5%, light intensity of 8,500 lx at midday, and photoperiod of 16 h light/8 h dark cycle. Microgreens were thus grown under natural conditions, with keeping environmental parameters monitored and recorded. Finally, theur ten-day-old microgreens were harvested.

### Determination of growth parameters

To evaluate growth, the fresh yield of one hundred de-rooted microgreens was determined. Also, microgreens were dried in an oven at 50℃ till a constant weight, and the dry yield of one hundred de-rooted microgreens was determined. Furthermore, microgreens were photographed alongside a ruler and subjected to digital image analysis using ImageJ software (version 1.34) to determine shoot length, root length, and leaf area. Following Hooshmand et al. [19], specific leaf area was calculated from Eq. (1):

1 $$Specific\:leaf\:area=\frac{Leaf\:area}{Leaf\:dry\:mass}$$

### Determination of physiological status

Photosynthetic pigments were extracted from fresh microgreens using dimethyl sulfoxide (DMSO). The extracts were read at 665 and 649 nm. Following Wellburn [20], chlorophyll a and chlorophyll b contents were calculated in µg mL^− 1^ from Eq. (2) and Eq. (3):

2 $$\begin{aligned}Chlorophyll\:a=&(12.19\:\times\:Absorbance\:at\:665)\\&-(3.45\:\times\:Absorbance\:at\:649)\end{aligned}$$

3 $$\begin{aligned}Chlorophyll\:b=&(21.99\:\times\:Absorbance\:at\:649)\\&-(5.32\:\times\:Absorbance\:at\:665)\end{aligned}$$

The pigment content was then converted into µg g^− 1^. Photosynthesis and transpiration rates were determined in situ immediately prior to harvest using a portable LCi gas exchange system (ADC BioScientific, UK) operated in open flow mode. Relative water content was determined following Schonfeld et al. [21] based on fresh weight (FW), dry weight (DW, obtained after oven-drying at 60℃ for 48 h), and turgid weight (TW, determined after water imbibition for 4 h) from Eq. (4):

4 $$\:Relative\:water\:content=\:\frac{FW-DW}{TW-DW}\:\times\:100$$

In addition, water use efficiency was calculated following Hooshmand et al. [19] as the ratio of total microgreen fresh yield per vessel to the cumulative volume of water consumed throughout the experiment period as derived from Eq. (5):

5 $$\:Water\:use\:efficiency=\:\frac{Microgreen\:fresh\:yield}{Volume\:of\:consumed\:water}$$

### Determination of stress biomarkers

Active osmolytes (proline, sodium, and chloride) were extracted from oven-dried microgreens using distilled water in a boiling water bath (3 times for an hour each). In contrast, catalase activity, lipid peroxidation, and hydrogen peroxide (H_2_O_2_) content were determined using fresh tissue samples. Proline content was spectrophotometrically determined at 515 nm using ninhydrin reagent [22]. Sodium concentration was determined via flame photometry, while chloride content was determined by silver nitrate titration method [23]. Catalase was extracted in 0.1 M phosphate buffer (pH 6.8) [24] and subsequently assayed by potassium permanganate titration [25]. Lipid peroxidation was spectrophotometrically determined at 532 and 600 nm as malondialdehyde (MDA) content using thiobarbituric acid method [26]. Also, H_2_O_2_ content was spectrophotometrically determined at 390 nm following potassium iodide method [27].

### Determination of proximate composition

Moisture content, ash content, total carbohydrates, total proteins, and total fats were determined according to AOAC [28] as described by Beshaw et al. [29]. Energy content was calculated using the Atwater factors according to USDA [30] from Eq. (6):

6 $$\begin{aligned}Energy\:content=&(4\:\times\:Total\:carbohydrates)\\&+(4\:\times\:Total\:proteins)\\&+(9\:\times\:Total\:fats)\end{aligned}$$

### Determination of mineral content

Following acid digestion of oven-dried microgreens with nitric acid, the elemental concentrations of potassium (K), calcium (Ca), magnesium (Mg), iron (Fe), manganese (Mn), and zinc (Zn) were determined via inductively coupled plasma optical emission spectroscopy (ICP-OES) using an iCAP 7400 spectrometer (Thermo Scientific, USA).

### Determination of fatty acid profile

Major saturated fatty acids (palmitic and stearic acid) as well as unsaturated fatty acids (oleic and linoleic acid) were determined via gas chromatography-mass spectrometry (GC-MS) analysis of their corresponding fatty acid methyl esters (FAMEs) using a TRACE 1310 GC-TSQ 9000 Triple Quadrupole MS (Thermo Scientific, USA). A direct capillary column TG-5MS (30 m × 0.25 mm × 0.25 μm film thickness) was used with helium gas as a carrier. The column flow was adjusted to 1 mL per minute. After collecting spectra, fatty acids were identified by comparison of their retention times and mass spectra with those of WILEY 09 and NIST 14 mass spectral databases. The total sums of saturated fatty acids (ΣSFA) and unsaturated fatty acids (ΣUFA) were subsequently computed.

### Determination of antioxidant capacity

Methanolic extracts obtained from oven-dried microgreens were used to determine total antioxidant activity, 2,2-diphenyl-1-picrylhydrazyl (DPPH)-scavenging activity, total phenols, and total flavonoids. Meanwhile, ascorbic acid and total carotenoids were determined in fresh tissue samples. Total antioxidant activity was determined following Prieto et al. [31], while DPPH-scavenging activity was determined following Siger et al. [32]. Total phenol content was determined following Spanos and Wrolstad [33], while total flavonoid content was determined following Shraim et al. [34]. Ascorbic acid content was determined following the titration method of Ogunlesi et al. [35], while total carotenoid content was determined according to Wellburn [20].

### Data processing and statistical analysis

Ten biological microgreens per treatment were randomly taken from the three vessel replicates to determine their growth parameters (*n* = 10). For the other investigations, de-rooted microgreens harvested from each vessel were pooled and homogenized separately to represent that specific vessel replicate (*n* = 3). Based on these replications, the mean values and standard deviations were tabulated. CoStat/CoHort software (version 6.311) was used to perform Tukey’s honestly significant difference (HSD) post-hoc test with one-way completely randomized analysis of variance (1WCR ANOVA). Accordingly, letters were used to denote significant differences at *p* ≤ 0.05. Thereafter, the statistical significance and coefficient of variation were indicated. Also, the percent change in each trait as affected by the different concentrations of NaCl was graphically represented as bar chart in the supplementary file. Collectively, the percent changes in all the concerned traits were plotted as a radar chart. Moreover, Past software (version 3.20) was used to conduct univariate and multivariate statistical analyses of the concerned traits and treatments. Pearson linear correlation analysis was applied as the univariate approach, while principal component analysis (PCA) and hierarchical cluster analysis (HCA) were performed as multivariate statistics.

## Results

### Effect of different NaCl concentrations on growth parameters

**Fig. 1:**
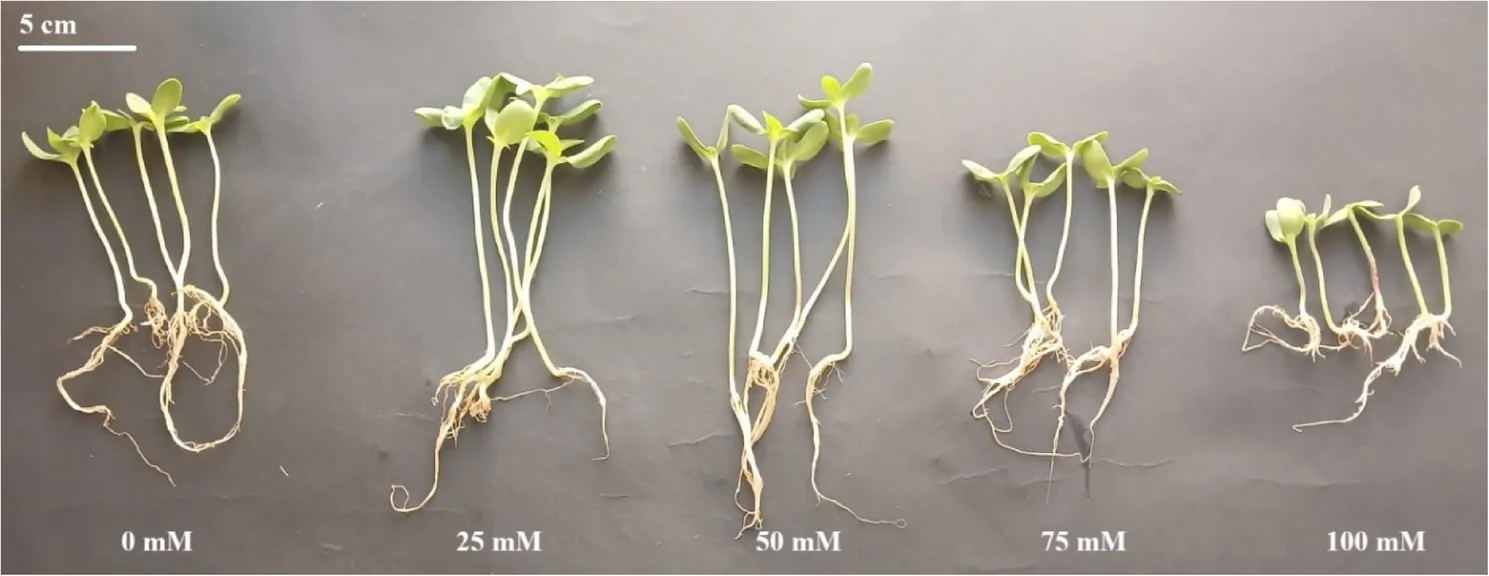
Effect of different NaCl concentrations (0, 25, 50, 75, and 100 mM) on ten-day-old sunflower microgreens

As shown in Fig. 1, Table 1 and Fig. S1, 25 and 50 mM NaCl significantly increased shoot fresh yield, dry yield, shoot length, root length, leaf area, and specific leaf area, with 25 mM NaCl being more effective than 50 mM. However, these growth parameters decreased by higher NaCl concentrations except for specific leaf area, which was non-significantly changed by all salt concentrations. A high level of statistical significance (*p* ≤ 0.001) was recorded for all the concerned growth parameters. The maximum coefficient of variation was observed for shoot fresh yield followed by root length.

**Table 1:** Effect of different NaCl concentrations (0, 25, 50, 75, and 100 mM) on growth parameters of sunflower microgreens

	Shoot fresh yield (g/100 microgreens)	Shoot dry yield (g/100 microgreens)	Shoot length (cm)	Root length (cm)	Leaf area (cm^2^)	Specific leaf area (cm^2^ g^–1^ DW)
0 mM	51.85b ± 10.64	3.25b ± 0.30	9.51c ± 1.07	9.30b ± 0.32	1.56c ± 0.14	69.03b ± 5.21
25 mM	82.31a ± 14.93	5.05a ± 0.53	14.10a ± 0.82	11.69a ± 0.97	2.83a ± 0.12	82.62a ± 3.45
50 mM	73.13a ± 13.19	5.02a ± 0.68	12.33b ± 0.40	10.25ab ± 1.51	2.07b ± 0.21	74.62ab ± 5.93
75 mM	52.47b ± 7.06	3.00bc ± 0.14	9.05c ± 1.19	7.64c ± 1.58	1.37d ± 0.09	78.84a ± 8.44
100 mM	36.24c ± 6.60	2.53c ± 0.26	6.03d ± 0.99	5.57d ± 1.48	0.93e ± 0.10	68.09b ± 10.86
Significance level	***	***	***	***	***	***
CV (%)	18.56	11.44	9.18	14.24	7.90	9.72

Data listed are means of ten replicates ± standard deviation. Different letters within a column refer to significant differences at *p* ≤ 0.05 based on Tukey’s test. Significance levels for treatment effects are indicated as *** (*p* ≤ 0.001), ** (*p* ≤ 0.01), and * (*p* ≤ 0.05). Coefficient of variation is referred to as CV

### Effect of different NaCl concentrations on physiological status

As shown in Table 2 and Fig. S2, 25 and 50 mM NaCl significantly increased chlorophyll a, chlorophyll b, photosynthesis rate, and transpiration rate, but non-significantly changed relative water content. Water use efficiency significantly increased by 25 mM NaCl, but non-significantly increased by 50 mM. However, most of these physiological parameters significantly decreased by higher NaCl concentrations. A high level of statistical significance (*p* ≤ 0.001) was recorded for all the concerned physiological traits. The maximum coefficient of variation was observed for photosynthesis rate followed by transpiration rate.

**Table 2:** Effect of different NaCl concentrations (0, 25, 50, 75, and 100 mM) on physiological status of sunflower microgreens

	Chlorophyll a (µg g^–1^ FW)	Chlorophyll b (µg g^–1^ FW)	Photosynthesis rate (µmol m^–2^ s^–1^)	Transpiration rate (mmol m^–2^ s^–1^)	Relative water content (%)	Water use efficiency (g L^–1^)
0 mM	238.29c ± 3.42	153.09b ± 4.27	2.28c ± 0.12	0.50c ± 0.03	78.75ab ± 0.27	65.84b ± 2.16
25 mM	289.04a ± 0.82	214.41a ± 2.85	4.26a ± 0.09	0.88a ± 0.04	80.81a ± 0.70	105.81a ± 3.06
50 mM	247.98b ± 3.07	207.75a ± 5.50	3.65b ± 0.07	0.72b ± 0.03	77.31bc ± 1.43	68.70b ± 0.72
75 mM	230.10d ± 1.73	157.68b ± 4.86	1.84d ± 0.30	0.48c ± 0.03	76.18c ± 0.53	57.66c ± 0.20
100 mM	163.65e ± 3.60	111.91c ± 5.48	1.06e ± 0.15	0.33d ± 0.03	69.44d ± 1.17	54.96c ± 0.05
Significance level	***	***	***	***	***	***
CV (%)	1.18	2.78	6.27	5.12	1.21	2.42

Data listed are means of three replicates ± standard deviation. Different letters within a column refer to significant differences at *p* ≤ 0.05 based on Tukey’s test. Significance levels for treatment effects are indicated as *** (*p* ≤ 0.001), ** (*p* ≤ 0.01), and * (*p* ≤ 0.05). Coefficient of variation is referred to as CV

### Effect of different NaCl concentrations on stress biomarkers

As shown in Table 3 and Fig. S3, 25 and 50 mM NaCl significantly increased proline content, while higher concentrations decreased it. For Na content, it gradually increased with increasing salt concentration. Meanwhile, Cl content was non-significantly changed by 25 and 50 mM NaCl, but significantly increased by higher salt concentrations. Catalase activity, lipid peroxidation, and H_2_O_2_ content were non-significantly changed by 25 mM. However, catalase activity significantly increased by 50 mM NaCl before decreasing by higher concentrations. Both lipid peroxidation and H_2_O_2_ content increased by concentrations higher than 25 mM. A high level of statistical significance (*p* ≤ 0.001) was recorded for all concerned stress biomarkers. The maximum coefficient of variation was observed for Cl.

**Table 3:** Effect of different NaCl concentrations (0, 25, 50, 75, and 100 mM) on stress biomarkers of sunflower microgreens

	Proline (mg g^–1^ DW)	Na (mmol g^–1^ DW)	Cl (mmol g^–1^ DW)	Catalase (unit g^–1^ FW)	Lipid peroxidation (µmol MDA g^–^¹ FW)	H_2_O_2_ (µmol g^–^¹ FW)
0 mM	4.53c ± 0.06	0.32e ± 0.01	1.46c ± 0.20	0.91bc ± 0.04	6.39c ± 0.07	46.92d ± 0.40
25 mM	4.79b ± 0.04	0.41d ± 0.01	1.92bc ± 0.20	0.97b ± 0.04	6.42c ± 0.03	47.53d ± 0.40
50 mM	5.56a ± 0.06	0.55c ± 0.01	2.14bc ± 0.39	1.16a ± 0.00	6.50c ± 0.04	48.84c ± 0.40
75 mM	4.44c ± 0.08	0.76b ± 0.01	2.70b ± 0.59	0.86c ± 0.04	10.43b ± 0.27	62.37b ± 0.39
100 mM	4.01d ± 0.04	0.96a ± 0.01	3.83a ± 0.20	0.70d ± 0.04	14.17a ± 0.31	74.76a ± 0.66
Significance level	***	***	***	***	***	***
CV (%)	1.25	1.67	14.48	3.82	2.12	0.83

*MDA*   malondialdehyde

Data listed are means of three replicates ± standard deviation. Different letters within a column refer to significant differences at *p* ≤ 0.05 based on Tukey’s test. Significance levels for treatment effects are indicated as *** (*p* ≤ 0.001), ** (*p* ≤ 0.01), and * (*p* ≤ 0.05). Coefficient of variation is referred to as CV

### Effect of different NaCl concentrations on proximate composition

As shown in Table 4 and Fig. S4, 25 mM NaCl non-significantly increased moisture content, while higher concentrations significantly decreased it. Conversely, 25 mM NaCl significantly decreased ash content, while higher concentrations significantly increased it. Also, 25 and 50 mM NaCl significantly increased total carbohydrates and total proteins, while higher concentrations non-significantly changed them. Conversely, 25 and 50 mM NaCl significantly decreased the level of total fats, while higher concentrations non-significantly changed them. For energy content, all salt concentrations non-significantly changed it. A high level of statistical significance (*p* ≤ 0.001) was recorded for all proximate traits, except for energy content that showed a low response (*p* ≤ 0.05). The maximum coefficient of variation was observed for total fats.

**Table 4:** Effect of different NaCl concentrations (0, 25, 50, 75, and 100 mM) on proximate composition of sunflower microgreens

	Moisture content (%)	Ash content (%)	Total carbohydrates (%)	Total proteins (%)	Total fats (%)	Energy content (Kcal/100 g)
0 mM	93.58a ± 0.19	1.72c ± 0.08	2.10c ± 0.22	1.66bc ± 0.06	0.94a ± 0.08	23.47ab ± 1.46
25 mM	93.67a ± 0.12	1.25d ± 0.05	2.68ab ± 0.04	2.11a ± 0.02	0.28b ± 0.16	21.70b ± 1.47
50 mM	91.50b ± 0.14	3.13b ± 0.19	2.92a ± 0.18	2.17a ± 0.11	0.27b ± 0.14	22.85b ± 1.52
75 mM	91.68b ± 0.29	3.54a ± 0.05	2.23c ± 0.10	1.61c ± 0.04	0.94a ± 0.23	23.82ab ± 2.48
100 mM	90.90c ± 0.08	3.74a ± 0.10	2.32bc ± 0.11	1.81b ± 0.05	1.24a ± 0.14	27.64a ± 1.06
Significance level	***	***	***	***	***	*
CV (%)	0.20	4.01	5.88	3.36	21.52	6.97

Data listed are means of three replicates ± standard deviation. Different letters within a column refer to significant differences at *p* ≤ 0.05 based on Tukey’s test. Significance levels for treatment effects are indicated as *** (*p* ≤ 0.001), ** (*p* ≤ 0.01), and * (*p* ≤ 0.05). Coefficient of variation is referred to as CV

### Effect of different NaCl concentrations on mineral content

As shown in Table 5 and Fig. S5, 25 mM NaCl significantly increased the level of Ca, Mg, Mn, and Zn, but significantly decreased the level of K and Fe. Higher salt concentrations either significantly or non-significantly decreased all these minerals. A high level of statistical significance (*p* ≤ 0.001) was recorded for all concerned minerals. The maximum coefficient of variation was observed for Fe followed by Ca.

**Table 5:** Effect of different NaCl concentrations (0, 25, 50, 75, and 100 mM) on mineral content of sunflower microgreens

	K (mg g^–1^ DW)	Ca (mg g^–1^ DW)	Mg (mg g^–1^ DW)	Fe (µg g^–1^ DW)	Mn (µg g^–1^ DW)	Zn (µg g^–1^ DW)
0 mM	11.92a ± 0.01	5.68b ± 0.35	5.37b ± 0.01	172.09a ± 11.78	23.02bc ± 0.01	114.83b ± 4.56
25 mM	10.50c ± 0.09	6.36a ± 0.01	6.08a ± 0.19	145.92bc ± 6.92	26.31a ± 1.05	129.60a ± 4.65
50 mM	11.59ab ± 0.33	4.85c ± 0.12	5.26b ± 0.16	162.35ab ± 2.06	23.82b ± 0.57	119.61b ± 1.54
75 mM	11.71ab ± 0.16	4.65c ± 0.21	4.98bc ± 0.16	133.64c ± 3.34	22.38bc ± 0.21	114.78b ± 2.93
100 mM	11.17bc ± 0.42	4.00d ± 0.06	4.75c ± 0.16	112.48d ± 0.45	22.05c ± 0.43	111.86b ± 2.19
Significance level	***	***	***	***	***	***
CV (%)	2.21	3.74	2.82	4.38	2.44	2.89

Data listed are means of three replicates ± standard deviation. Different letters within a column refer to significant differences at *p* ≤ 0.05 based on Tukey’s test. Significance levels for treatment effects are indicated as *** (*p* ≤ 0.001), ** (*p* ≤ 0.01), and * (*p* ≤ 0.05). Coefficient of variation is referred to as CV

### Effect of different NaCl concentrations on fatty acid profile

As shown in Table 6 and Fig. S6, 25 mM NaCl significantly decreased the relative percentage of palmitic, stearic, and oleic acid, but significantly increased linoleic acid. Thus, 25 mM NaCl significantly decreased total saturated fatty acids, but non-significantly increased total unsaturated fatty acids. Higher salt concentrations increased palmitic, stearic, and total saturated fatty acids, but they mostly decreased linoleic, oleic, and total unsaturated fatty acids. A high level of statistical significance (*p* ≤ 0.001) was recorded for all concerned fatty acids, except for total unsaturated fatty acids that showed a moderate response (*p* ≤ 0.01). The maximum coefficient of variation was observed for stearic acid.

**Table 6:** Effect of different NaCl concentrations (0, 25, 50, 75, and 100 mM) on fatty acid profile (relative %) of sunflower microgreens

	Palmitic acid (%)	Stearic acid (%)	Linoleic acid (%)	Oleic acid (%)	ΣSFA (%)	ΣUFA (%)
0 mM	6.88c ± 0.01	2.31c ± 0.14	48.69b ± 0.29	42.12b ± 0.01	9.19d ± 0.15	90.81a ± 0.31
25 mM	6.21d ± 0.01	2.03d ± 0.02	51.12a ± 0.97	40.10c ± 0.02	8.24e ± 0.02	91.22a ± 0.95
50 mM	7.33b ± 0.27	2.46bc ± 0.02	48.10bc ± 0.61	41.85b ± 0.38	9.79c ± 0.26	89.95ab ± 0.98
75 mM	9.39a ± 0.08	2.58b ± 0.06	46.86c ± 0.48	40.59c ± 0.35	11.97b ± 0.14	87.45c ± 0.67
100 mM	9.72a ± 0.10	2.82a ± 0.10	43.10d ± 0.49	45.33a ± 0.63	12.54a ± 0.13	88.43bc ± 1.12
Significance level	***	***	***	***	***	**
CV (%)	1.68	3.43	1.28	0.86	1.53	0.95

*ΣSFA  * total saturated fatty acids, *ΣUFA  * total unsaturated fatty acids

Data listed are means of three replicates ± standard deviation. Different letters within a column refer to significant differences at *p* ≤ 0.05 based on Tukey’s test. Significance levels for treatment effects are indicated as *** (*p* ≤ 0.001), ** (*p* ≤ 0.01), and * (*p* ≤ 0.05). Coefficient of variation is referred to as CV

### Effect of different NaCl concentrations on antioxidant capacity

As shown in Table 7 and Fig. S7, 25 and 50 mM NaCl significantly increased total antioxidant activity, DPPH-scavenging activity, and total phenols, while higher salt concentrations significantly decreased these traits. However, most salt concentrations non-significantly changed total flavonoids. Also, all salt concentrations non-significantly changed ascorbic acid, except for 25 mM NaCl that significantly increased it. For total carotenoids, 25 mM NaCl significantly increased it, while higher salt concentrations significantly or non-significantly decreased it. A high level of statistical significance (*p* ≤ 0.001) was recorded for all concerned antioxidant traits. The maximum coefficient of variation was observed for ascorbic acid.

**Table 7:** Effect of different NaCl concentrations (0, 25, 50, 75, and 100 mM) on antioxidant capacity of sunflower microgreens

	Total antioxidant activity (mg AAE mL^–1^)	DPPH-scavenging activity (%)	Total phenols (mg GAE g^–1^ DW)	Total flavonoids (mg QE g^–1^ DW)	Ascorbic acid (mg g^–1^ FW)	Total carotenoids (µg g^–1^ FW)
0 mM	0.85c ± 0.03	91.88c ± 0.39	55.93c ± 0.53	1.03ab ± 0.04	0.65bc ± 0.06	43.94b ± 2.55
25 mM	1.17b ± 0.01	97.44a ± 0.11	60.87b ± 0.23	0.94b ± 0.03	0.89a ± 0.03	49.00a ± 2.39
50 mM	1.26a ± 0.01	95.26b ± 0.29	61.95a ± 0.34	1.00ab ± 0.05	0.68b ± 0.04	37.85c ± 0.55
75 mM	0.59d ± 0.02	80.21d ± 1.13	53.88d ± 0.08	0.77c ± 0.02	0.60bc ± 0.13	41.83bc ± 0.78
100 mM	0.46e ± 0.01	91.07c ± 0.66	33.99e ± 0.43	1.05a ± 0.02	0.46c ± 0.05	31.20d ± 0.70
Significance level	***	***	***	***	***	***
CV (%)	1.76	0.68	0.67	3.52	10.77	4.05

*AAE  * ascorbic acid equivalent, *DPPH  * 2,2-diphenyl-1-picrylhydrazyl, *GAE  * gallic acid equivalent, *QE  * quercetin equivalent

Data listed are means of three replicates ± standard deviation. Different letters within a column refer to significant differences at *p* ≤ 0.05 based on Tukey’s test. Significance levels for treatment effects are indicated as *** (*p* ≤ 0.001), ** (*p* ≤ 0.01), and * (*p* ≤ 0.05). Coefficient of variation is referred to as CV

### Correlations among the concerned traits

**Fig. 2:**
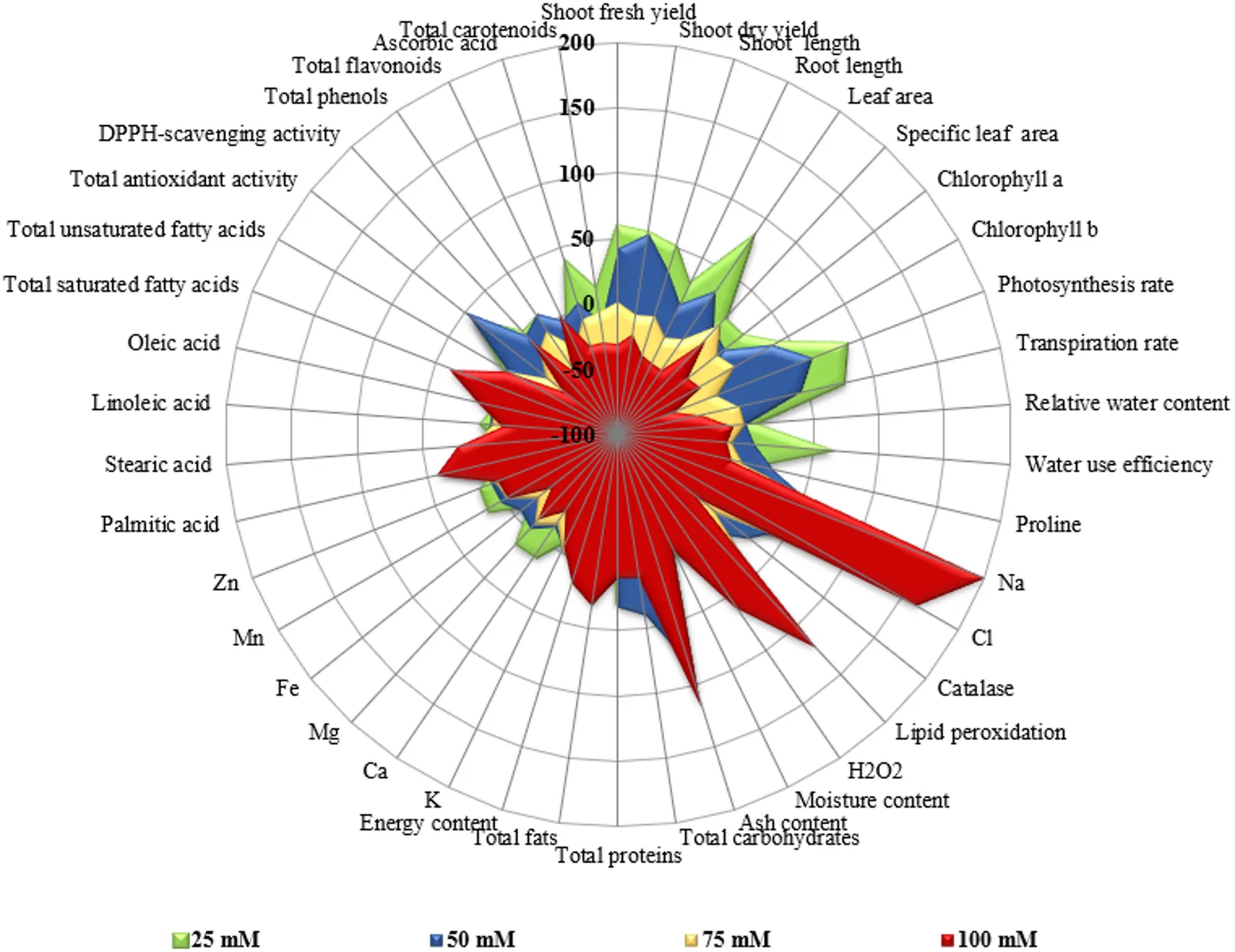
Radar chart of the percent change in the concerned traits of sunflower microgreens as affected by different NaCl concentrations (25, 50, 75, and 100 mM) compared with control (0 mM NaCl)

**Fig. 3:**
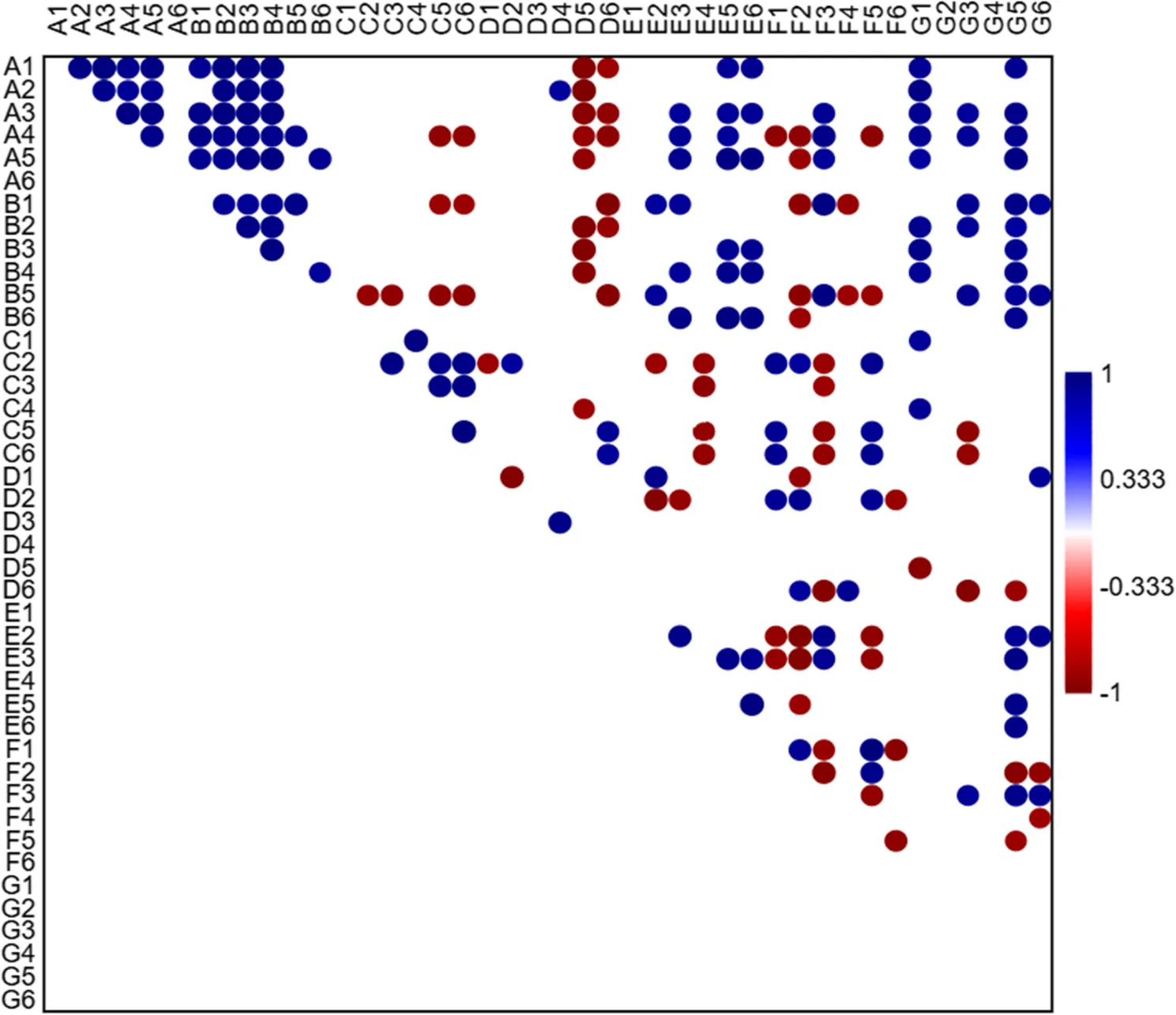
heatmap of linear correlation coefficients (r) among the concerned traits of sunflower microgreens as affected by different NaCl concentrations. Only significant correlations at *p* ≤ 0.05 are represented as colored bubbles. (A1 = shoot fresh yield, A2 = shoot dry yield, A3 = shoot length, A4 = root length, A5 = leaf area, A6 = specific leaf area, B1 = chlorophyll a, B2 = chlorophyll b, B3 = photosynthesis rate, B4 = transpiration rate, B5 = relative water content, B6 = water use efficiency, C1 = proline, C2 = Na, C3 = Cl, C4 = catalase, C5 = lipid peroxidation, C6 = H_2_O_2_, D1 = moisture content, D2 = ash content, D3 = total carbohydrates, D4 = total proteins, D5 = total fats, D6 = energy content, E1 = K, E2 = Ca, E3 = Mg, E4 = Fe, E5 = Mn, E6 = Zn, F1 = palmitic acid, F2 = stearic acid, F3 = linoleic acid, F4 = oleic acid, F5 = total saturated fatty acids, F6 = total unsaturated fatty acids, G1 = total antioxidant activity, G2 = DPPH-scavenging activity, G3 = total phenols, G4 = total flavonoids, G5 = ascorbic acid, G6 = total carotenoids)

Regarding growth parameters, Figs. 2 and 3 showed that shoot fresh yield, shoot dry yield, shoot length, and root length positively correlated with each other, and with chlorophyll a, chlorophyll b, photosynthesis rate, and transpiration rate. Separately, root length positively correlated with relative water content but correlated negatively with lipid peroxidation and H_2_O_2_ content. Also, leaf area positively correlated with water use efficiency. In addition, shoot fresh yield, shoot dry yield, shoot length, root length, and leaf area negatively correlated with total fats, while most of these traits positively correlated with Mg, Mn, and Zn. Furthermore, shoot length, root length, and leaf area positively correlated with linoleic acid. Moreover, most of the considered growth parameters positively correlated with total antioxidant activity, total phenols, and ascorbic acid.

For physiological status, Fig. 3 showed that chlorophyll a, chlorophyll b, photosynthesis rate, and transpiration rate positively correlated with each other. Meanwhile, chlorophyll a negatively correlated with lipid peroxidation and H_2_O_2_ content. Moreover, most of these physiological traits alongside relative water content and water use efficiency positively correlated with minerals (particularly Ca, Mg, Mn, and Zn) and also with antioxidant capacity (particularly total antioxidant activity, total phenols, ascorbic acid, and total carotenoids). For stress biomarkers, proline positively correlated with catalase activity and total antioxidant activity. Also, Na positively correlated with Cl, lipid peroxidation, H_2_O_2_ content, and ash content, but negatively correlated with moisture content, Ca, and Mg. In addition, Cl positively correlated with lipid peroxidation and H_2_O_2_ content, but negatively correlated with Fe. Moreover, catalase activity positively correlated with total antioxidant activity; and lipid peroxidation positively correlated with H_2_O_2_ content.

Regarding proximate composition, moisture content positively correlated with Ca and total carotenoids, while ash content positively correlated with Na, and negatively correlated with Ca, Mg, and moisture content. For minerals, Ca positively correlated with Mg, linoleic acid, ascorbic acid, and total carotenoids, but negatively correlated with palmitic acid, stearic acid, and total saturated fatty acids. Mg positively correlated with Mn, Zn, linoleic acid, and ascorbic acid, but negatively correlated with palmitic acid, stearic acid, and total saturated fatty acids. Mn positively correlated with Zn and ascorbic acid, but negatively correlated with stearic acid. Zn positively correlated with ascorbic acid. Regarding fatty acid profile, palmitic acid positively correlated with stearic acid and total saturated fatty acids, but negatively correlated with linoleic acid and total unsaturated fatty acids. Stearic acid negatively correlated with linoleic acid, total unsaturated fatty acids, ascorbic acid, and total carotenoids. Linoleic acid positively correlated with total phenols, ascorbic acid, and total carotenoids, but negatively correlated with total saturated fatty acids. In addition, total saturated fatty acids negatively correlated with total unsaturated fatty acids.

### Principal component analysis

**Fig. 4:**
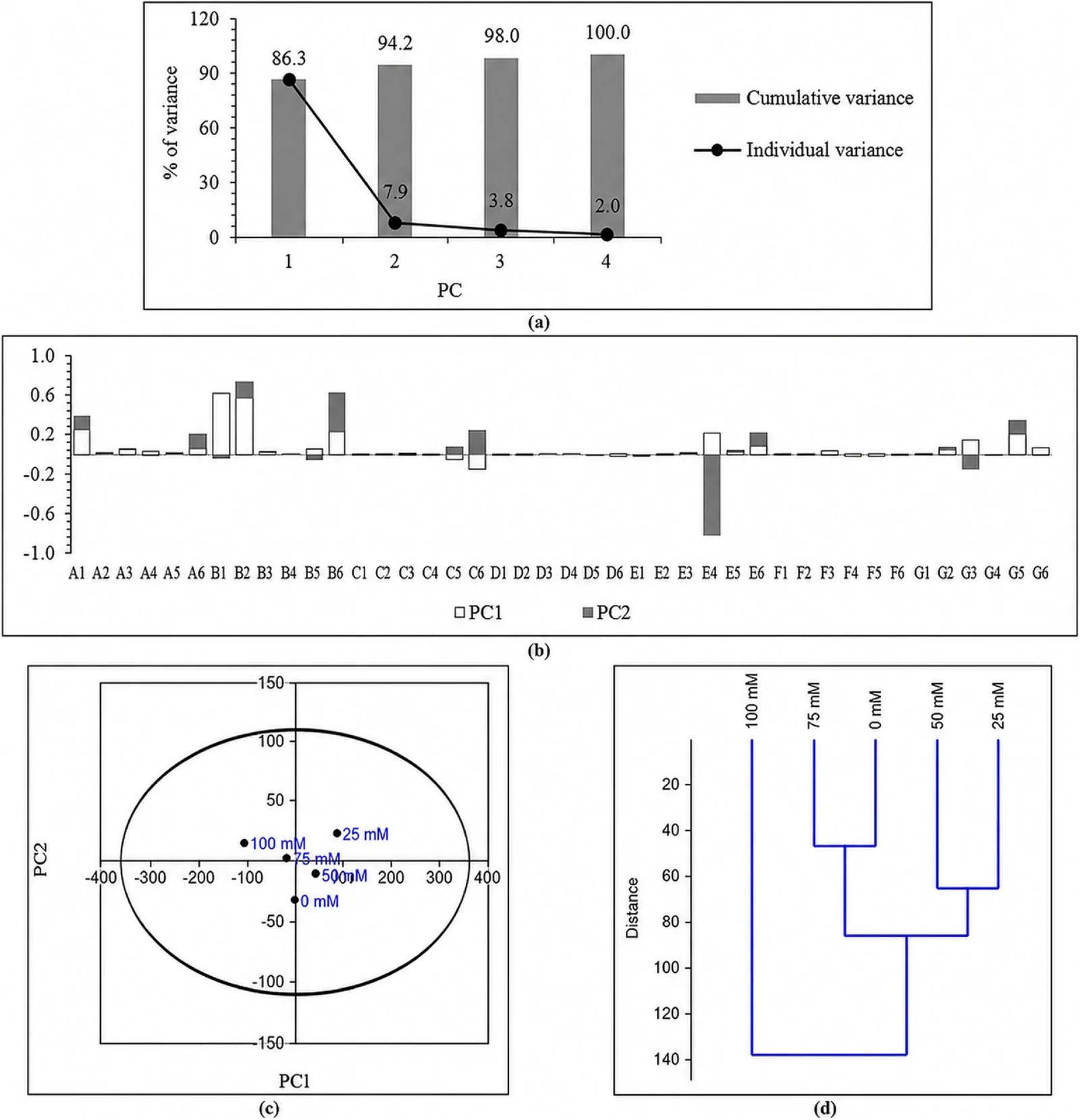
Multivariate analysis based on the concerned traits of sunflower microgreens as affected by different NaCl concentrations (**a**: scree plot of the principal components (PCs), **b**: loading plot of the PCs, **c**: principal component analysis of the five NaCl treatments, **d**: hierarchical cluster analysis of the five NaCl treatments). (A1 = shoot fresh yield, A2 = shoot dry yield, A3 = shoot length, A4 = root length, A5 = leaf area, A6 = specific leaf area, B1 = chlorophyll a, B2 = chlorophyll b, B3 = photosynthesis rate, B4 = transpiration rate, B5 = relative water content, B6 = water use efficiency, C1 = proline, C2 = Na, C3 = Cl, C4 = catalase, C5 = lipid peroxidation, C6 = H_2_O_2_, D1 = moisture content, D2 = ash content, D3 = total carbohydrates, D4 = total proteins, D5 = total fats, D6 = energy content, E1 = K, E2 = Ca, E3 = Mg, E4 = Fe, E5 = Mn, E6 = Zn, F1 = palmitic acid, F2 = stearic acid, F3 = linoleic acid, F4 = oleic acid, F5 = total saturated fatty acids, F6 = total unsaturated fatty acids, G1 = total antioxidant activity, G2 = DPPH-scavenging activity, G3 = total phenols, G4 = total flavonoids, G5 = ascorbic acid, G6 = total carotenoids)

As shown in Fig. 4a, PCA revealed four principal components (PCs) that accounted for the total variance. The first component (PC1) explained 86.3% of variance, while PC1 and PC2 together explained 94.2% of variance. As shown in Fig. 4b and Table S1, PC1 was primarily driven by positive loadings from chlorophyll a, chlorophyll b, shoot fresh yield, water use efficiency, Fe, and ascorbic acid. Conversely, PC1 was primarily driven by negative loading from H_2_O_2_ content. For PC2, it was primarily driven by positive loadings from water use efficiency, H_2_O_2_ content, specific leaf area, and chlorophyll b. Conversely, PC2 was primarily driven by negative loadings from Fe and total phenol.

As shown in Fig. 4c and Table S2, PCA score plot distinguished the five NaCl treatments based on the concerned traits of sunflower microgreens. The control (0mM NaCl) treatment was placed in the lower region of the plot, characterized by a minor negative PC1 score and a prominent negative PC2 score. Moderate stress (50 mM NaCl) caused a slight shift in almost the same region. However, low salt stress (25 mM NaCl) caused a sharp shift into the upper right quadrate with the maximum scores. High and severe stress (75 and 100 mM) caused another marked shift leftward, characterized by a negative PC1 score and a minor positive PC2 score for 75 mM NaCl, as well as a prominent negative PC1 score and a positive PC2 score for 100 mM NaCl.

### Hierarchical cluster analysis

As shown in Fig. 4d, HCA separated the five NaCl treatments into two main clusters based on the concerned traits of sunflower microgreens. The first cluster consisted only of the 100 mM NaCl treatment. The second cluster included the other four treatments and was further sub-divided into two subgroups. One subgroup paired the 75 and 0 mM treatments, while the other subgroup paired the 50 and 25 mM treatments.

## Discussion

The current study demonstrates that NaCl salinity can exert a dual effect on sunflower microgreens. Regarding growth parameters, 25 and 50 mM NaCl maximized shoot yield, structural elongation, and leaf expansion. Thus, these salinity levels can be considered as eustress factors, with 25 mM recorded as the optimal threshold. Conversely, 75 and 100 mM NaCl caused typical distress for most growth parameters. However, the increase or non-significant change in specific leaf area by all salt treatments reveals a morphological plasticity of the microgreens. Matching our findings, Plocek et al. [11] recorded that salt stress induced by 11 mM NaCl significantly increased the fresh weight of broccoli microgreens and the dry weight of purslane microgreens. Also, Wang et al. [36] observed that 40 mM NaCl significantly increased the length and leaf area of broccoli sprouts. Moreover, Melo et al. [37] found that the specific leaf area of *Calotropis* plants increased under 100 mM NaCl stress.

The decline observed in growth parameters under 75 and 100 mM NaCl marks the transition from eustress to distress. This inhibitory effect is caused by osmotic stress and ion toxicity. Elevated NaCl concentrations create an osmotic barrier that restricts root ability to absorb water. This water deficit reduces cell turgor pressure and inhibits cell division and enlargement. Also, the accumulation of ions within the plant cells disrupts ion homeostasis through the competitive inhibition of essential ions such as potassium. Excessive ions also induce oxidative stress by the accumulation of ROS [38].

The biphasic growth responses of sunflower microgreens to salt treatments can be ascribed to the alterations in their physiological and biochemical behavior. Based on statistical correlations, modified growth is closely related to chlorophyll content as well as photosynthesis and transpiration rates. Also, the altered growth attributes heavily rely on mineral uptake, as indicated by the positive correlations with essential minerals like Mg, Mn, and Zn. In addition, the recorded alterations in growth vigor can be linked with the antioxidant defense, as supported by the positive correlations of most growth parameters with total antioxidant activity, total phenols, and ascorbic acid. Conversely, total fats stand as a major negative correlate, meaning that resource allocation shifts away from fat accumulation when biomass production and elongation are prioritized. Regarding leaf expansion, it is associated with enhanced biomass production and minimal water loss, as indicated by the positive correlation between leaf area and water use efficiency. Moreover, root elongation appears to be restricted by oxidative stress, as indicated by the negative correlation of root length with lipid peroxidation and H_2_O_2_ content.

Regarding physiological status, low and mild NaCl concentrations improved the photosynthetic machinery and leaf gas exchange capacity of sunflower microgreens. Also, 25 mM NaCl proved to be the most effective treatment. This salinity level significantly increased chlorophyll a, chlorophyll b, photosynthesis rate, transpiration rate, and water use efficiency, and non-significantly increased relative water content. Although 50 mM NaCl maintained a stimulatory effect, its magnitude was lower than that of 25 mM. In contrast, these physiological parameters significantly decreased under 75 and 100 mM NaCl. Matching our findings, Wang et al. [36] reported that 40 and 80 mM NaCl enhanced photosynthetic rate in broccoli sprouts before inhibitory effects appeared at higher NaCl levels. Also, Ma et al. [38] found that 150 mM NaCl reduced photosynthetic rate and transpiration rate in cotton plants. Similarly, Hussain et al. [39] showed that 100 mM NaCl maintained photosynthetic performance in blue panic grass close to the control, while 300 mM NaCl caused marked inhibition.

The increase in chlorophyll content under low-to-mild salinity suggests that these levels enhanced the light-harvesting capacity of the microgreens. The marked increase in chlorophyll b may reflect an adaptive adjustment in antenna complexes associated with photosystem II [40]. Also, the increased photosynthesis rate that accompanies higher pigment content suggests that the accumulated pigments were effectively translated into higher carbon assimilation. The positive correlations among chlorophyll a, chlorophyll b, photosynthesis rate, and transpiration rate further support these interpretations. In this context, evaluation of pigment and gas exchange attributes is well documented to reveal the regulation of several photosynthetic processes [41]. Furthermore, the enhancement of these physiological parameters under eustress can be directly linked to the increase in mineral content, as most of these parameters positively correlated with Ca, Mg, Mn, and Zn. This correlation is supported by Ohnishi et al. [42], who documented the importance of these minerals for photosynthesis and chlorophyll biosynthesis.

Also, the increase in transpiration rate indicates that low-to-mild salinity did not impose stomatal limitation. Instead, these treatments maintained sufficient stomatal opening to enhance CO_2_ diffusion into the leaf mesophyll and support the observed increase in photosynthetic rate. However, the increase in transpiration rate was accompanied by a relative water content close to the control. This stability in leaf water status suggests that the microgreens could maintain tissue hydration through efficient water uptake and osmotic adjustment. Moreover, the significant increase in water use efficiency under 25 mM NaCl may indicate that the gain in carbon assimilation exceeded the cost of water loss. Meanwhile, the non-significant increase in water use efficiency under 50 mM NaCl suggests that the balance between photosynthesis and transpiration became less favorable.

Conversely, the decline in these physiological traits under higher salinity levels can be attributed to the combined effects of osmotic stress and ionic toxicity. According to Wang et al. [43], salt stress negatively affects chloroplast structure and function through osmotic, ionic, and oxidative stresses. This can be supported by the negative correlation recorded herein for chlorophyll a with lipid peroxidation and H_2_O_2_ content, alongside the positive correlation of most of these physiological parameters with antioxidant capacity. In addition, the stronger reduction in photosynthesis compared with transpiration at 100 mM NaCl suggests that both stomatal and non-stomatal limitations were involved in suppressing carbon assimilation. The involvement of both limitations in suppressing photosynthesis under salt stress was recorded elsewhere [44].

With respect to stress biomarkers, low and mild salinity enhanced proline accumulation and catalase activity, alongside non-significant change in lipid peroxidation and H_2_O_2_ content. The higher proline content observed under 25 and 50 mM NaCl suggests that the microgreens activated osmoprotective mechanisms in response to eustress. Proline accumulation is a well-known adaptive response to salt stress because it contributes to osmotic adjustment, stabilizes proteins and membranes, protects cellular structures, and participates in ROS scavenging [45]. However, the reduction in proline under 75 and 100 mM NaCl may reflect impaired proline biosynthesis, enhanced proline degradation, or the consumption of proline in stress defense processes [46].

In addition, Na and Cl gradually accumulated as salinity increased. At low levels of salinity, this ion accumulation may contribute to osmotic adjustment [47]. However, at higher levels, their excessive accumulation can disturb ionic homeostasis and impair membrane stability [48]. The change in catalase activity, H_2_O_2_ content, and lipid peroxidation provides strong evidence for the transition from biochemical acclimation to oxidative injury. Under 50 mM NaCl, catalase activity increased, suggesting an efficient activation of enzymatic antioxidant defense against H_2_O_2_. This activation likely helped restrict oxidative damage, as H_2_O_2_ and lipid peroxidation increased but very slightly. In contrast, catalase activity decreased under 75 and 100 mM NaCl, while H_2_O_2_ content and lipid peroxidation increased. This indicates that moderate-to-severe salinity caused ROS accumulation beyond the scavenging capacity of the antioxidant system. The positive correlation between lipid peroxidation, Na, and H₂O₂ content further supports this interpretation. Under severe salinity, excessive ion accumulation enhances ROS formation, and the resulting H₂O₂ can oxidize membrane lipids, leading to MDA production. Thus, the combined increase in Na, Cl, H_2_O_2_, and MDA, together with the decline in catalase, reflects a shift from defense to oxidative damage [47].

Matching our results, Plocek et al. [11] reported that salinity eustress increased proline accumulation in broccoli microgreens. The ionic response observed in this study is also supported by Hussain et al. [39], who showed that NaCl can act as a cheap osmoticum under low salinity, supporting osmotic adjustment and water uptake, while excessive ion uptake under high salinity can cause ion toxicity or imbalance. Moreover, Beisekova et al. [46] working on barley showed that salinity increased oxidative stress indicators, including H_2_O_2_ and MDA; and that the changes in proline accumulation were closely related to the level of oxidative stress.

For proximate composition, 25 mM NaCl non-significantly increased moisture content, while higher salinity levels significantly decreased it. This indicates the plant ability to maintain osmotic homeostasis and trigger water uptake despite the rise in transpirational water loss. This can be supported by the stability of relative water content under the same salinity level. Meanwhile, the significant decrease in moisture content at higher salinity levels can result from osmotic stress and physiological drought. Matching these findings, Abd Elkader et al. [49] found that 34 mM NaCl stress increased the moisture content of radish microgreens. Also, Plocek et al. [11] recorded that low salinity increased the moisture content of broccoli microgreens.

Conversely, 25 mM NaCl significantly decreased ash content of sunflower microgreens, while higher salinity levels significantly increased it. These findings demonstrate a negative correlation between moisture and ash content. As will be discussed later, low salinity significantly enhanced the accumulation of Ca, Mg, Mn, and Zn, while it decreased K and Fe contents. Then, the significant reduction in K at 25 mM NaCl can be the primary cause of the overall drop in ash content. Because K is typically the most abundant cation in plant tissues [50], a sharp drop in K can heavily decrease total ash content. Although higher salinity levels decreased K, Ca, Mg, Fe, Mn, and Zn, the overall ash content increased, more likely due to the massive accumulation of Na and Cl. This interpretation can be confirmed by the strong positive correlation between ash content and Na. Matching our findings, Abd Elkader et al. [49] recorded that NaCl eustress caused a marked decrease in ash content of radish microgreens.

In addition, 25 and 50 mM NaCl increased total carbohydrates and total proteins in sunflower microgreens. This accumulation of primary metabolites under low and mild salinity is a well-documented osmotic adjustment strategy, where plants synthesize soluble sugars and nitrogenous compounds to maintain cellular turgor [51]. Matching these results, Abd Elkader et al. [49] observed that 34 mM NaCl increased total carbohydrates and proteins in radish microgreens. The accumulation of carbohydrates recorded herein under low and mild salinity can be attributed to the significant enhancement in the plant photosynthetic efficiency. This aligns with our earlier observation where these levels increased chlorophyll content, which directly resulted in a remarkable increase in photosynthesis rate.

Nevertheless, this metabolic shift of sunflower microgreens toward osmotic defense appears to occur at the expense of lipid biosynthesis, as indicated by the significant decrease in total fats under 25 and 50 mM NaCl treatments. This lipid down-regulation matches the trend observed by El-Gebaly et al. [52] on fenugreek sprouts, where 34 mM NaCl reduced total fat content. Furthermore, and despite these substantial shifts in primary metabolites, the total energy content of sunflower microgreens remained non-significantly changed across all salt concentrations. This aligns with the stable caloric observations reported by Farhat et al. [53] in cress plants under salt stress.

Regarding mineral composition, 25 mM NaCl enhanced the accumulation of Ca, Mg, Mn, and Zn in sunflower microgreens. Because microgreens were grown on water only, this altered mineral profile may reflect the accelerated mobilization, translocation, and retention of internal seed reserves from the cotyledons to the developing shoot. Thus, the increase in these elements can be regarded as a defense mechanism, where low osmotic stress upregulates the breakdown of cotyledon reserves to maintain cellular turgor and metabolic balance. In addition, the mineral composition of sunflower microgreens closely correlated with their physiological performance and antioxidant status. The enhanced accumulation of Mg supports the recorded increase in chlorophyll content, while increased Ca content can preserve membrane stability. Also, the increased availability of Mn and Zn can provide essential cofactors for antioxidant enzymes. Conversely, 25 mM NaCl reduced K and Fe content. In absence of external nutrients, the reduction in K content can be attributed to internal ion antagonism and cellular leaking. Furthermore, accumulated Na may cause a targeted K leakage out of the cells [54]. Moreover, it was formerly postulated that the reduction in Fe content was linked to the salinity-induced blocking or down-regulation of Fe-regulated transporter proteins required to remobilize Fe stores from the cotyledons during early germination [55].

As salinity increased to mild and moderate levels, these beneficial effects disappeared, leading to severe damage across the whole plant at 100 mM NaCl. At these higher thresholds, concentrations of K, Ca, Mg, Fe, Mn, and Zn decreased. The excessive accumulation of intracellular Na and Cl causes osmotic stress, which limits water absorption and reduces tissue hydraulic conductivity [56]. Accordingly, the transport of essential elements like Ca and Mg from cotyledon reserves to the growing shoot may be impaired. Furthermore, high salinity triggers the accumulation of H_2_O_2_ and causes membrane lipid peroxidation. This oxidative stress can damage cellular structures, decrease catalase activity, and lead to the leakage of minerals out of the cell.

In accordance with our findings, Voutsinos-Frantzis et al. [57] found that salt stress induced by 20 mM NaCl reduced the leaf accumulation of K and Fe in corn salad plants. Also, Ullah et al. [58] observed that 34 mM NaCl stress increased Ca and Mn contents in duckweed plants. Also, Abd Elkader et al. [49] demonstrated that 34 mM NaCl stress enhanced the accumulation of Ca and Zn in radish microgreens. Moreover, Loupassaki et al. [59] recorded that 25 mM NaCl stress increased Mg accumulation in certain olive cultivars before declining at higher salinity levels.

Regarding fatty acid profile, 25 mM NaCl significantly decreased palmitic, stearic, and oleic acid, with a significant increase in linoleic acid. Consequently, low salinity induced a significant decrease in total saturated fatty acids alongside a non-significant increase in total unsaturated fatty acids. This metabolic regulation indicates that low salinity induces an adaptive mechanism to increase membrane fluidity and help the plant maintain cellular function. The depletion of palmitic, stearic, and oleic acids may refer to the rapid upregulation of fatty acid desaturases that convert saturated precursors such as palmitic acid and stearic acid into monounsaturated oleic acid. The latter is then converted into polyunsaturated linoleic acid. Enhanced lipid unsaturation at low stress levels is a well-documented strategy by which the plant cells maintain membrane fluidity and prevent structural damage [60].

However, higher salt concentrations increased palmitic, stearic, and total saturated fatty acids, but decreased linoleic, oleic, and total unsaturated fatty acids. The decline in polyunsaturated fatty acids at elevated NaCl concentrations may be driven by the inhibition of desaturases due to ion toxicity and oxidative stress. Rapid ROS generation under severe salinity can attack the double bonds of polyunsaturated fatty acids through lipid peroxidation, resulting in degradation products such as malondialdehyde [61]. Nonetheless, the increase recorded herein in saturated fatty acids can be a trial to limit ion leakage through the damaged membranes. Under stress, plant cells tend to accumulate saturated fatty acids to increase membrane rigidification, which reduces membrane permeability to toxic ions but restricts essential nutrient and water transport [62].

These interpretations of the biphasic fatty acid shift are supported by correlations with other traits. The breakdown of membrane integrity under high salinity level is confirmed by the positive correlations of palmitic acid, stearic acid, and total saturated fatty acids with Na accumulation, lipid peroxidation, and H_2_O_2_ content. Conversely, the protective role of polyunsaturated fatty acids is demonstrated by the negative correlations of linoleic acid with these stress markers. This statistical contrast shows that when antioxidants like flavonoids and ascorbic acid fail to scavenge H_2_O_2_, lipid peroxidation breaks down the fluid linoleic acid. This is supported by the positive correlation between linoleic acid and these antioxidants. As a result, the accumulation of rigid saturated fatty acids limits cellular elongation and development, which is validated by the positive correlations of linoleic acid and negative correlations of total saturated fatty acids with some growth parameters like shoot length and root length.

Our findings align with previous studies showing that lipids in sunflower seedling cotyledons are primarily composed of oleic and linoleic acids, followed by palmitic and stearic acids [63]. Matching the changes recorded herein, Gogna et al. [63] found that on day six of salt stress, a salt-tolerant sunflower variety exhibited an increase in linoleic and stearic acid, alongside a reduction in palmitic and oleic acid. Also, Giménez et al. [64] recorded that purslane microgreens responded to salinity with an increase in linoleic acid and concurrent reductions in stearic and oleic acid.

For antioxidant-related compounds and activities, low-to-mild NaCl salinity enhanced total antioxidant activity, DPPH-scavenging activity, and total phenolic content. In this respect, 50 mM NaCl was the most effective treatment for enhancing total antioxidant activity and phenolic accumulation. Meanwhile, 25 mM NaCl was more favorable for ascorbic acid and total carotenoids. This threshold-dependent response agrees with the concept that controlled salinity may stimulate secondary metabolism, while excessive salinity becomes inhibitory [65]. The increase in total antioxidant activity under 25 and 50 mM NaCl appears to be mainly associated with the stimulation of non-enzymatic antioxidant metabolism, particularly phenolic compounds. Phenolics are important secondary metabolites that contribute to ROS scavenging, membrane protection, and cellular redox balance under abiotic stress [66]. Also, ascorbic acid is a key antioxidant involved in ROS detoxification. Moreover, carotenoids help protect the photosynthetic apparatus from excess excitation energy. However, total flavonoids were mostly non-significantly changed, suggesting that flavonoid accumulation was not the main pathway responsible for the enhanced antioxidant activity under the present conditions.

The decline in antioxidant capacity under 75 and 100 mM NaCl indicates that moderate-to-severe salinity exceeded the beneficial eustress range. At 75 mM NaCl, total antioxidant activity, DPPH-scavenging activity, total phenols, total flavonoids, ascorbic acid, and total carotenoids decreased. This reduction can be explained by osmotic stress, ion toxicity, and oxidative damage, which disturb cellular metabolism, suppress phenolic-related biosynthesis, damage chloroplasts, accelerate carotenoid degradation, and deplete antioxidant pools under excessive ROS production [67].

In agreement with our findings, Islam et al. [68] reported that controlled NaCl salinity improved bioactive compounds and antioxidant activity in wheat microgreens. In their study, 12.5 mM NaCl maximized β-carotene, phenolic acid, flavonoid, and ascorbic acid contents. They also found that 25 mM NaCl produced the highest DPPH-scavenging activity. Also, Pavlović et al. [65] found that cauliflower microgreens grown under 50 mM NaCl increased phenol and flavonoid content, whereas the response at 100 mM NaCl was weaker or more variable.

Based on PCA results, it can be concluded that the major differences among the five salt treatments are driven by chlorophyll accumulation, biomass yield, plant water status, as well as the enrichment of essential minerals (Fe) and secondary metabolites (ascorbic acid). Also, it is indicated that the resilience of sunflower microgreens to salinity is coupled with their capacity to lower H_2_O_2_ content. More importantly, PCA reveals a hormetic response of sunflower microgreens to salinity. Low salt stress effectively acted as a eustress that shifted their metabolic profile toward maximum biomass yield, nutritional value, and antioxidant activity. Mild stress caused a transitional phase where metabolic cost of salt adaptation enforced the plant performance back near the control level. However, moderate and severe stress induced marked distress that restricted biomass yield and the overall performance of microgreens. This was further validated by HCA that isolated the 100 mM NaCl treatment from the other four treatments. This confirms that severe salinity induced a distinct distress. The moderate stress treatment was paired with the control but near the severe stress treatment. This confirms that moderate salinity also brought about marked distress. Meanwhile, the mild stress treatment was paired with the low stress which came at the most first position. This confirms that low salinity level is the most effective eustress for sunflower microgreens.

## Conclusions

Sunflower microgreens show a clear threshold-dependent response to salinity. Low stress induced by 25 mM NaCl acts as an optimal eustress primer to maximize their nutritional value before higher salinity levels cause detrimental distress. Physiologically, low salt stress triggers cellular defense through osmotic regulation without inducing oxidative damage. Conversely, exceeding 50 mM NaCl causes growth inhibition, ion imbalance, and marked oxidative stress. The core scientific contribution of this work is thus establishing the precise threshold where salinity transitions from a metabolic elicitor to a destructive stressor in an oilseed microgreen. Therefore, low salt stress can be re-purposed in controlled agriculture as a simple, low-cost, and chemical-free tool to grow high-value functional food. However, further molecular studies are crucial to unravel the underlying mechanisms. Also, it is vital in future studies to determine the exact amounts of antinutritional factors in response to eustress.

## Supplementary Information


Supplementary Material 1.


## Data Availability

All data supporting the findings of this study are available within the paper and its Supplementary Information.
