# Supplementary material for: Salt eustress modulates physiological responses and enhances yield, nutritional quality, and antioxidant capacity in sunflower microgreens
Source: BMC Plant Biol. 2026 Aug 27;26:1483. doi: 10.1186/s12870-026-09786-y (PMC13523218; doi:10.1186/s12870-026-09786-y)
Supplement: Supplementary file 1 [file 12870_2026_9786_MOESM1_ESM.docx]

**Figure S1.** Percent change in growth parameters of sunflower microgreens as affected by different NaCl concentrations (25, 50, 75, and 100 mM) compared with control (0 mM NaCl). Asterisk refers to significant difference from control at *p* ≤ 0.05 based on Tukey’s test.

**Figure S2.** Percent change in physiological status of sunflower microgreens as affected by different NaCl concentrations (25, 50, 75, and 100 mM) compared with control (0 mM NaCl). Asterisk refers to significant difference from control at *p* ≤ 0.05 based on Tukey’s test.

**Figure S3.** Percent change in stress biomarkers of sunflower microgreens as affected by different NaCl concentrations (25, 50, 75, and 100 mM) compared with control (0 mM NaCl). Asterisk refers to significant difference from control at *p* ≤ 0.05 based on Tukey’s test.

**Figure S4.** Percent change in proximate composition of sunflower microgreens as affected by different NaCl concentrations (25, 50, 75, and 100 mM) compared with control (0 mM NaCl). Asterisk refers to significant difference from control at *p* ≤ 0.05 based on Tukey’s test.

**Figure S5.** Percent change in minerals content of sunflower microgreens as affected by different NaCl concentrations (25, 50, 75, and 100 mM) compared with control (0 mM NaCl). Asterisk refers to significant difference from control at *p* ≤ 0.05 based on Tukey’s test.

**Figure S6.** Percent change in fatty acids profile of sunflower microgreens as affected by different NaCl concentrations (25, 50, 75, and 100 mM) compared with control (0 mM NaCl). Asterisk refers to significant difference from control at *p* ≤ 0.05 based on Tukey’s test.

**Figure S7.** Percent change in antioxidant capacity of sunflower microgreens as affected by different NaCl concentrations (25, 50, 75, and 100 mM) compared with control (0 mM NaCl). Asterisk refers to significant difference from control at *p* ≤ 0.05 based on Tukey’s test.

**Table S1.** Loading plot of principal components (PCs) based on the concerned traits of sunflower microgreens as affected by different NaCl concentrations

| **Trait** | | **PC1** | **PC2** |
| --- | --- | --- | --- |
| Growth parameters | Shoot fresh yield | **0.25** | 0.14 |
|  | Shoot dry yield | 0.01 | 0.01 |
|  | Shoot length | 0.04 | 0.01 |
|  | Root length | 0.03 | -0.01 |
|  | Leaf area | 0.01 | 0.01 |
|  | Specific leaf area | 0.06 | **0.15** |
| Physiological status | Chlorophyll a | **0.62** | -0.03 |
|  | Chlorophyll b | **0.57** | **0.15** |
|  | Photosynthesis rate | 0.02 | 0.01 |
|  | Transpiration rate | 0.00 | 0.00 |
|  | Relative water content | 0.06 | -0.05 |
|  | Water use efficiency | **0.24** | **0.38** |
| Stress biomarkers | Proline | 0.01 | -0.01 |
|  | Na | 0.00 | 0.01 |
|  | Cl | -0.01 | 0.02 |
|  | Catalase | 0.00 | 0.00 |
|  | Lipid peroxidation | -0.04 | 0.07 |
|  | H_2_O_2_ | **-0.15** | **0.24** |
| Proximate composition | Moisture content | 0.01 | -0.01 |
|  | Ash content | -0.01 | 0.01 |
|  | Total carbohydrates | 0.00 | 0.00 |
|  | Total proteins | 0.00 | 0.00 |
|  | Total fats | -0.01 | 0.00 |
|  | Energy content | -0.03 | 0.02 |
| Minerals content | K | 0.00 | -0.02 |
|  | Ca | 0.01 | 0.00 |
|  | Mg | 0.01 | 0.00 |
|  | Fe | **0.21** | **-0.81** |
|  | Mn | 0.02 | 0.03 |
|  | Zn | 0.09 | **0.13** |
| Fatty acids profile | Palmitic acid | -0.02 | 0.02 |
|  | Stearic acid | 0.00 | 0.00 |
|  | Linoleic acid | 0.04 | -0.01 |
|  | Oleic acid | -0.02 | 0.00 |
|  | Total saturated fatty acids | -0.02 | 0.02 |
|  | Total unsaturated fatty acids | 0.02 | -0.01 |
| Antioxidant capacity | Total antioxidant activity | 0.00 | 0.00 |
|  | DPPH-scavenging activity | 0.04 | 0.03 |
|  | Total phenols | 0.15 | **-0.15** |
|  | Total flavonoids | 0.00 | 0.00 |
|  | Ascorbic acid | **0.21** | 0.13 |
|  | Total carotenoids | 0.08 | -0.01 |

**Table S2.** Treatment scores plot of principal components (PCs) based on the concerned traits of sunflower microgreens as affected by different NaCl concentrations

| **Treatment** | PC1 | PC2 |
| --- | --- | --- |
| 0 mM | -1.93 | -31.65 |
| 25 mM | 85.48 | 23.46 |
| 50 mM | 41.02 | -9.79 |
| 75 mM | -18.83 | 2.72 |
| 100 mM | -105.74 | 15.26 |
